# Target value of mean arterial pressure in patients undergoing continuous renal replacement therapy due to acute kidney injury

**DOI:** 10.1186/s12882-020-02227-4

**Published:** 2021-01-09

**Authors:** Yaerim Kim, Donghwan Yun, Soie Kwon, Kyubok Jin, Seungyeup Han, Dong Ki Kim, Kook-Hwan Oh, Kwon Wook Joo, Yon Su Kim, Sejoong Kim, Seung Seok Han

**Affiliations:** 1 Department of Internal Medicine, Keimyung University School of Medicine, Daegu, Korea; 2 Department of Internal Medicine, Seoul National University College of Medicine, 103 Daehakro, Jongno-gu, Seoul, 03080 Korea; 3 Department of Internal Medicine, Seoul National University Bundang Hospital, 82, Gumi-ro 173beon-gil, Bundang-gu, Seongnam-si, Gyeonggi-do 13620 Korea

**Keywords:** Acute kidney injury, Continuous renal replacement therapy, Mean arterial pressure, Mortality

## Abstract

**Background:**

Although patients undergoing continuous renal replacement therapy (CRRT) due to acute kidney injury (AKI) frequently have instability in mean arterial pressure (MAP), no consensus exists on the target value of MAP related to high mortality after CRRT.

**Methods:**

A total of 2,292 patients who underwent CRRT due to AKI in three referral hospitals were retrospectively reviewed. The MAPs were divided into tertiles, and the 3^rd^ tertile group served as a reference in the analyses. The major outcome was all-cause mortality during the intensive care unit period. The odds ratio (OR) of mortality was calculated using logistic regression after adjustment for multiple covariates. The nonlinear relationship regression model was applied to determine the threshold value of MAP related to increasing mortality.

**Results:**

The mean value of MAP was 80.7 ± 17.3 mmHg at the time of CRRT initiation. The median intensive care unit stay was 5 days (interquartile range, 2–12 days), and during this time, 1,227 (55.5%) patients died. The 1^st^ tertile group of MAP showed an elevated risk of mortality compared with the 3^rd^ tertile group (adjusted OR, 1.28 [1.03–1.60]; *P* = 0.029). In the nonlinear regression analysis, the threshold value of MAP was calculated as 82.7 mmHg. Patients with MAP < 82.7 mmHg had a higher mortality rate than those with ≥ 82.7 mmHg (adjusted OR, 1.21 [1.01–1.45]; *P* = 0.037).

**Conclusions:**

Low MAP at CRRT initiation is associated with a high risk of mortality, particularly when it is < 82.7 mmHg. This value may be used for risk classification and as a potential therapeutic target.

**Supplementary Information:**

The online version contains supplementary material available at 10.1186/s12882-020-02227-4.

## Background

Preventing hypotension is a crucial issue in critically ill patients because of its relationship with high mortality and other poor outcomes [1, 2]. Accordingly, the guidelines for septic patients requiring vasopressors suggest initially maintaining mean arterial pressure (MAP) > 65 mmHg, followed by monitoring via multiple hemodynamic parameters to an endpoint of tissue perfusion [3]. Furthermore, this target value should be individualized to the pertinent circumstances or patient characteristics.

Continuous renal replacement therapy (CRRT) is a rescue therapy for patients displaying both unstable vital signs and severe acute kidney injury (AKI). Hypotension after initiating CRRT is a frequent occurrence [4] and can aggravate CRRT-related mortality, reaching 50% [5, 6]. Because patients requiring CRRT have AKI as well as other critical conditions, their target value of MAP may be different from that of other critically ill patient subsets or recommended MAP values. An elegant multicenter study including patients with septic shock suggests that a MAP of 65–70 mmHg is enough to guarantee survival compared with 80–85 mmHg [7]; however, only 40% of those patients had AKI, and there was no information regarding CRRT. The target blood pressure values in hemodialysis patients are suggested as < 140 mmHg and < 90 mmHg for systolic and diastolic blood pressures, respectively (i.e., MAP is calculated as 106.7 mmHg) [8], but this value could not be applied in cases of CRRT because the patient condition is much different from that of conventional hemodialysis patients. Collectively, there are no current guidelines or evidence for the target value of MAP in patients initiating CRRT due to AKI. Therefore, we addressed this issue using a multicenter cohort of patients with AKI requiring CRRT.

## Methods

### Study populations

A total of 2,292 patients who were ≥ 18 years old and had AKI requiring CRRT were consecutively reviewed from January 2010 to December 2019. Of these, patients with underlying end-stage renal disease before starting CRRT (*n* = 81) were excluded. Accordingly, 2,211 patients were analyzed in the study (Figure S1). Under the terms of ethics approval, the requirement for informed consent was waived.

### Study variables and outcome

The data at the time of CRRT initiation were obtained via electronic medical records. Clinical data such as age, sex, body weight, application of mechanical ventilation, use of vasopressors and comorbidities including diabetes mellitus, hypertension, ischemic heart disease, chronic obstructive pulmonary disease, and cancer were reviewed. The vital signs, such as the mean arterial pressure, heart rate, respiratory rate, body temperature, and alveolar–arterial oxygen difference, were measured. Blood laboratory data, such as pH, white blood cell count, hemoglobin, platelets, sodium, potassium, blood urea nitrogen, creatinine, albumin, and bilirubin, were measured. There were no missing variables. The Acute Physiologic Assessment and Chronic Health Evaluation (APACHE) II score was calculated based on the method presented in the original study [9]. The primary outcome was all-cause mortality during the stay in the intensive care unit (ICU).

### Statistical analysis

Statistical analyses were performed using SPSS (version 23.0; IBM Corp., Armonk, NY, USA), SAS (version 9.4; SAS Institute Inc., Cary, NC, USA), and R (version 3.5.3; The Comprehensive R Archive Network: http://cran.r-project.org) software. Categorical and continuous variables were expressed as proportions and means ± standard deviations if they had a normal distribution and as medians with interquartile ranges if they were not normally distributed. The chi-square test was used for comparison of categorical variables (Fisher’s exact test if applicable), and Student’s t-test or the Mann-Whitney *U* test was used for continuous variables with or without a normal distribution, respectively. Initially, patients were divided according to the tertiles of MAP, and the risk of ICU mortality was calculated using the logistic regression model with stepwise multivariate adjusting methods. The variance inflation factor was less than 1.5 for all variables, which indicated there was no collinearity. Additive generalized models with penalized splines (package: *mgcv*) were used to analyze the relationship between MAP and ICU mortality. A target value was indicated when the lower limit of 95% confidence intervals of ORs was ≥ 0 compared with a reference MAP. Kaplan-Meier survival curves were drawn and compared using the log rank test. *P* values < 0.05 were defined as significant when they were set to two-sided.

## Results

### Study subjects

The baseline characteristics of the patients are shown in Table 1. The mean age of the patients was 65.0 ± 15.2 years, and 60.8% were male. The CRRT mode was continuous veno-venous hemodiafiltration. The median duration of CRRT was 3 days (IQR, 1–7 days). The mean value of MAP at the time of CRRT initiation was 80.7 ± 17.3 mmHg. When the patients were divided according to the tertiles of MAP, the lowest tertile group displayed older age, lower values of arterial pH, serum hemoglobin, creatinine, and albumin, and higher scores for APACHE II than the other tertiles (Table 1).

**Table 1:** Baseline characteristics according to the mean arterial pressure

Variables	Total (*n* = 2,211)	1^st^ tertile (*n* = 746)	2^nd^ tertile (*n* = 724)	3^rd^ tertile (*n* = 741)	*P*-value
Age (year)	65.0 ± 15.2	66.4 ± 14.7^‡^	64.9 ± 15.6	63.6 ±15.1	0.001
Male (%)	60.8	58.6	60.4	63.6	0.137
Body weight (kg)	60.9 ±12.7	60.5 ± 12.5*	60.3 ± 12.8*	61.9 ± 12.8	0.034
Mechanical ventilator (%)	79.1	80.0	79.0	78.3	0.705
Vasopressors (%)	69.0	70.1	69.5	67.5	0.520
Diabetes mellitus (%)	27.1	34.8	26.7	26.7	0.802
Hypertension (%)	26.5	28.2	23.3	27.9	0.063
Ischemic heart disease (%)	11.0	11.8	10.6	10.5	0.687
COPD (%)	3.7	2.9	4.4	3.6	0.324
Cancer (%)	34.3	34.7	35.6	32.5	0.434
Mean arterial pressure (mmHg)	80.7 ± 17.3	63.3 ± 8.3^‡^	79.3 ± 3.9^‡^	99.7 ± 11.9	< 0.001
Heart rate (/min)	104.7 ± 25.9	103.4 ± 26.4	104.9 ± 24.8	105.7 ± 26.4	0.235
Respiratory rate (/min)	23.5 ± 7.8	23.4 ± 7.9	23.3 ± 7.8	23.7 ± 7.7	0.646
Body temperature (°C)	36.5 ± 1.5	36.4 ± 1.9	36.5 ± 1.3	36.5 ±1.2	0.093
FiO2	0.6 [0.4–0.9]	0.6 [0.4–1.0]	0.6 [0.4–0.9]	0.6 [0.4–0.9]	0.172
PaO2	92.0 [71.0–128.0]	89.5 [70.0–124.0]	96.5 [73.0–133.0]	90.0 [69.8–128.3]	0.311
AaDO_2_	253.7 [122.4–469.0]	284.6 [132.9–499.9]	241.6 [119.7–454.1]	243.3 [119.2–471.1]	0.161
Laboratory findings					
pH	7.3 ± 0.2	7.3 ± 0.1^†^	7.3 ± 0.1	7.3 ± 0.1	0.003
White blood cells (×10^3^/uL)	11.9 [6.7–18.7]	11.6 [6.5–19.5]	11.9 [7.0–18.6]	12.2 [6.7–18.3]	0.609
Hemoglobin (g/dL)	9.8 ± 2.1	9.7 ± 2.0*	9.7 ± 2.2*	10.0 ± 2.1	0.019
Platelet (×10^3^/uL)	102.0 [59.0–166.0]	98.0 [59.0–157.0]	103.0 [58.0–171.0]	105.0 [58.0–171.0]	0.906
Sodium (mmol/L)	138.5 ± 7.6	138.8 ± 8.0	138.1 ± 7.5	138.5 ± 7.2	0.154
Potassium (mmol/L)	4.4 ± 1.0	4.4 ± 1.0	4.3 ± 0.9	4.4 ± 1.0	0.313
BUN (mg/dL)	44.0 [29.0–65.0]	41.0 [27.0–64.0]	45.5 [30.0–66.0]	45.0 [30.0–65.0]	0.243
Creatinine (mg/dL)	2.4 [1.6–3.5]	2.2 [1.6–3.3]*	2.4 [1.6–3.6]	2.5 [1.6–3.7]	0.031
Albumin (g/dL)	2.8 ± 0.6	2.7 ± 0.6^‡^	2.8 ± 0.6*	2.9 ± 0.6	< 0.001
Bilirubin (mg/dL)	1.8 [0.8–4.1]	1.9 [0.9–4.1]	1.9 [0.8–4.5]	1.6 [0.8–3.7]	0.935
CRRT dose (ml/kg/hr)	40.0 [35.0–42.0]	40.0 [35.0–43.8]	40.0 [35.0–44.0]	40.0 [35.0–40.0]	0.402
APACHE II	22.4 ± 9.7	24.6 ± 9.8^‡^	21.1 ± 9.5	21.3 ± 9.5	< 0.001

All continuous variables were represented with median [interquartile range]

*COPD* Chronic obstructive pulmonary disease, *MAP* mean arterial pressure, *AaDO*_*2*_ alveolar-arterial oxygen difference, *BUN* blood urea nitrogen, *CRRT* continuous renal replacement therapy, *APACHE* Acute Physiology and Chronic Health Evaluation

*P < 0.05; ^†^*P* < 0.01; ^‡^*P* < 0.001, compared with the 3^rd^ tertile

### Relationship between MAP and mortality

**Fig. 1:**
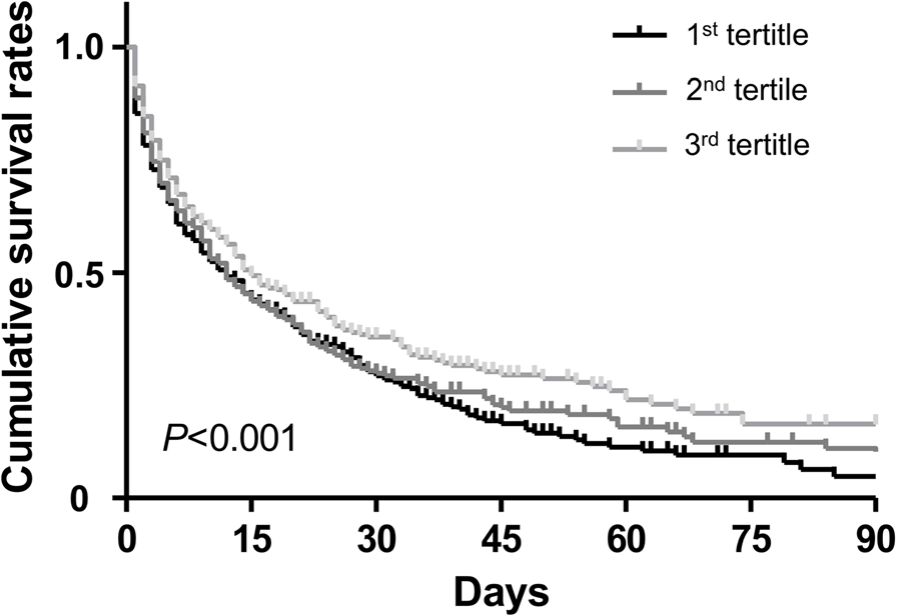
Survival curves according to the tertiles of mean arterial pressure

The association between MAP levels and ICU mortality was evaluated after categorizing patients into the tertiles of MAP. A total of 1,227 patients (55.5%) died during the ICU period. The prevalence of death was 61.1%, 55.4%, and 49.9% from the 1^st^ to the 3^rd^ tertiles. In the univariate analysis, the 1^st^ tertile group had a 58% increased risk for mortality compared with the 3^rd^ tertile group (Table 2). Even after several variables were adjusted in a stepwise fashion, the 1^st^ tertile group had a higher risk of mortality than the 3^rd^ tertile group (Table 2). The Kaplan-Meier survival curves also supported these results (Fig. 1).

**Table 2:** Risk of intensive care unit-mortality according to the tertiles of mean arterial pressure

	Model 1		Model 2		Model 3		Model 4	
Groups	OR (95% CI)	*P*-value	OR (95% CI)	*P*-value	OR (95% CI)	*P*-value	OR (95% CI)	*P*-value
3^rd^ tertile	1 (Reference)		1 (Reference)		1 (Reference)		1 (Reference)	
2^nd^ tertile	1.245 (1.014–1.529)	0.037	1.253 (1.020–1.540)	0.031	1.210 (0.974–1.504)	0.086	1.183 (0.949–1.475)	0.136
1^st^ tertile	1.577 (1.283–1.937)	< 0.001	1.596 (1.298–1.963)	< 0.001	1.342 (1.076–1.673)	0.009	1.290 (1.031–1.614)	0.026

Model 1: Not adjusted

Model 2: Adjusted for age and sex

Model 3: Model 2 plus, body weight, body temperature, using inotropics, support by mechanical ventilator, serum pH, serum hemoglobin, serum albumin, and APACHE II score

Model 4: Model 3 plus variables with *P* < 0.1 in univariate analysis

*CI* confidence interval, *OR* odds ratio

### Target value of MAP related to high mortality

**Fig. 2:**
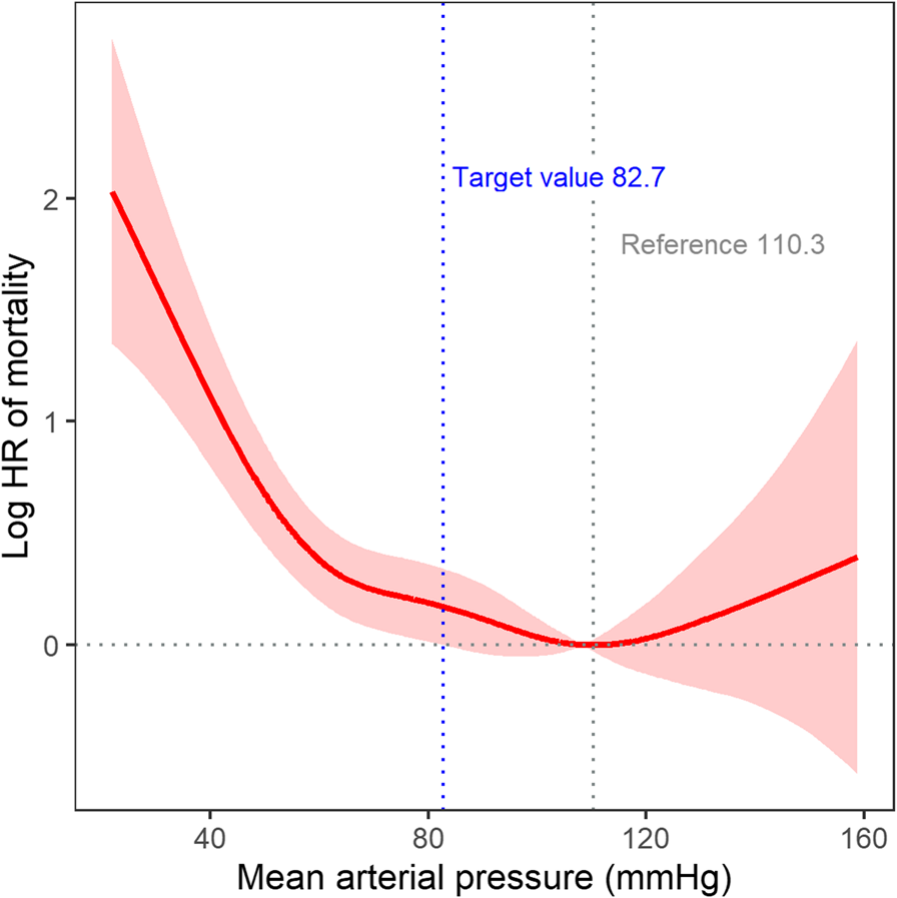
Nonlinear relationship between mean arterial pressure and mortality. The area indicates 95% confidence intervals. HR, hazard ratio

The nonlinear relationship between MAP and the risk of ICU mortality demonstrated that there seemed to be a target value of MAP related to increasing mortality (Fig. 2). To identify the target value, we used the penalized spline curve with a reference of which log hazard ratio (HR) and the 95% confidence interval were set to zero, and a referential MAP was defined when the minimal log HR was between 80 mmHg and 100 mmHg. When MAP was 82.7 mmHg, log HR started to increase, and its lower 95% confidence interval was > 0. Accordingly, 82.7 mmHg appeared to be the target value of MAP for ICU mortality.

**Fig. 3:**
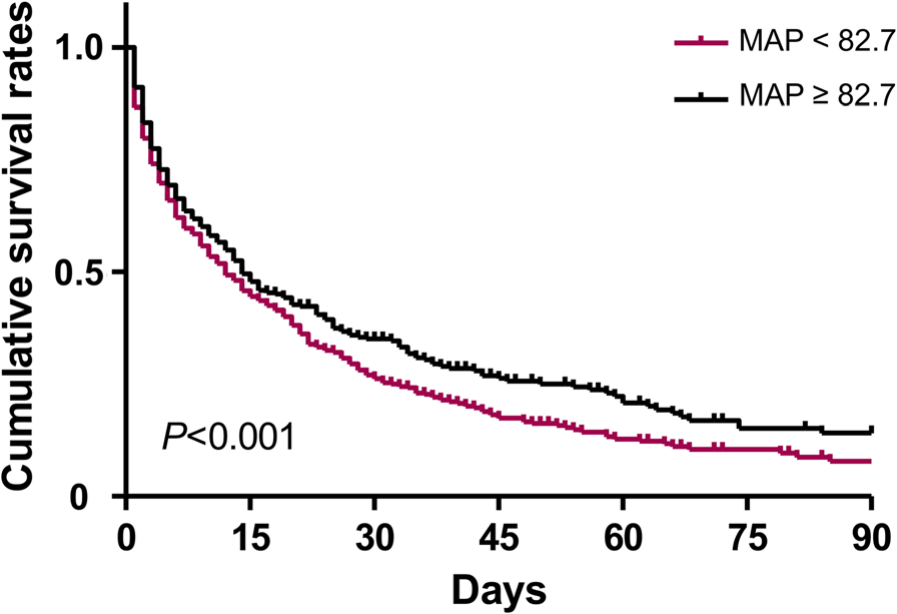
Survival during the stay of the intensive care unit according to the high (≥ 82.7 mmHg) and low (< 82.7 mmHg) mean arterial pressures (MAPs)

**Fig. 4:**
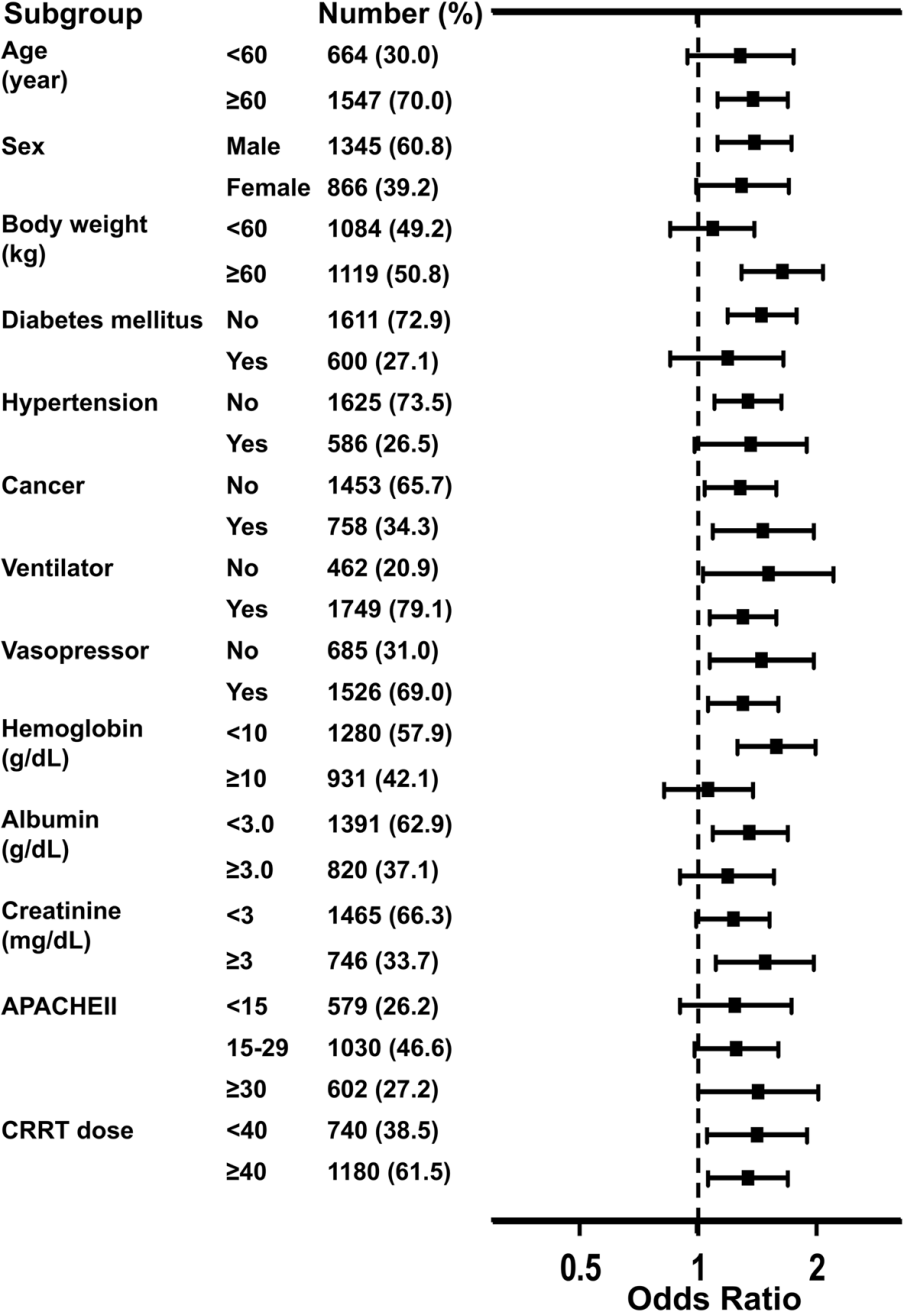
Odds ratios of mortality in the low mean arterial pressure group (< 82.7 mmHg) compared with the high mean arterial pressure group (≥ 82.7 mmHg) according to the patient status. APACHE, Acute Physiology and Chronic Health Evaluation

After dividing patients into high (≥ 82.7 mmHg) and low (< 82.7 mmHg) MAP groups, the low MAP group had different characteristics from the high MAP group based on age; sex; body weight; heart rate; serum values of hemoglobin, creatinine, and albumin; and APACHE II score (Table S1). Patients with low MAP had an additional 34.4% risk of ICU mortality compared with those with high MAP (Table 3). After several variables were adjusted in a stepwise fashion, the low MAP group was associated with high ICU mortality (Table 3). The Kaplan-Meier survival curves also supported these results (Fig. 3). Figure 4 demonstrates that the high risk of ICU mortality depending on the target value of MAP remained consistent irrespective of age, sex, diabetes mellitus, and other variables.

**Table 3:** Risk of intensive care unit-mortality in patients with low mean arterial pressure

	Model 1		Model 2		Model 3		Model 4	
Groups	OR (95% CI)	*P*-value	OR (95% CI)	*P*-value	OR (95% CI)	*P*-value	OR (95% CI)	*P*-value
High MAP (≥ 82.7 mmHg)	1 (Reference)		1 (Reference)		1 (Reference)		1 (Reference)	
Low MAP (< 82.7 mmHg)	1.344 (1.134–1.593)	0.001	1.354 (1.141–1.605)	0.001	1.219 (1.017–1.462)	0.032	1.222 (1.015–1.471)	0.034

Model 1: Not adjusted

Model 2: Adjusted for age and sex

Model 3: Model 2 plus, body weight, body temperature, using inotropics, support by mechanical ventilator, serum pH, serum hemoglobin, serum albumin, and APACHE II score

Model 4: Model 3 plus variables with *P* < 0.1 in univariate analysis

*CI* confidence interval, *MAP* mean arterial pressure, *OR* odds ratio

## Discussion

Prevention of hypotension and maintaining proper tissue perfusion is the treatment goal for critically ill patients. Alarming a target value of MAP should facilitate the management of these patients, but this issue has not been evaluated in patients requiring CRRT despite their high mortality rates. The present study identified that baseline MAP at the time of starting CRRT was associated with the mortality risk. When a nonlinear relationship was applied, the potential target value of MAP was calculated as 82.7 mmHg. Accordingly, a warning for MAP values above this target may be needed at the time of initiating CRRT.

Among the patients requiring intensive care, a substantial proportion of patients suffered from AKI and may have needed the support of CRRT. AKI adds a significant burden to critically ill patients and ultimately leads to a poor outcome and high mortality. Among several risk factors in this patient subset, an abnormal blood pressure range has been included in the scoring system of outcome prediction [9–11]. Although CRRT provides gentle modification of electrolyte, acid-base, and fluid imbalance with hemodynamic tolerance compared with intermittent hemodialysis, early exposure to hypotension during CRRT may be associated with high mortality [6]. In addition to tissue hypoxia, low blood pressures affect the adequacy of CRRT by clotting in the circuit system. This event finally propagates hypotension as a vicious cycle. Accordingly, increasing the blood pressures over the target value may be needed before starting CRRT as a preventive management.

MAP levels of less than 60 or 70 mmHg were associated with mortality in patients with septic shock [12, 13]. Based on these results, the Surviving Sepsis Guideline suggests that the cutoff value of MAP in septic patients is 65 mmHg [14], which was supported by a cohort study wherein MAP less than 65 mmHg with a longer duration of exposure significantly increased the mortality of patients with septic shock [15]. A large randomized trial showed that a higher target for MAP of 80 to 85 mmHg did not differ from 65 to 70 mmHg regarding the risk of mortality in patients with septic shock [7]. Another randomized trial, in which a higher target for MAP of 75 to 80 mmHg did not provide a survival benefit compared with 60 to 65 mmHg in patients with shock regardless of the cause, supports these results [16]. The target value may be individualized according to the patient characteristics and comorbidities. For example, higher targets are needed in patients with atherosclerosis and/or previous hypertension than in young patients without cardiovascular comorbidity [14]. However, this issue has not been resolved in patients with AKI requiring CRRT until the present study. The present target value of 82.7 mmHg is higher than the 65 mmHg recommended by the guidelines [7, 12–17]. Patients with AKI requiring CRRT are in poor medical condition with impairment of the autoregulatory system [18, 19], which requires higher blood circulation than patients without severe AKI. These results do not justify the use of excessive inotropics in patients with low MAP because of their side effects. The issue on the inotropics use should be solved in the future study with randomized, controlled design.

Although the present study had strengths, such as a large number of CRRT patients from multi-centers, there were certain limitations to be discussed. The study could not determine causality between MAP and mortality because of the nature of its retrospective design. The important confounders, such as heart function, cause of AKI, severity of AKI at the time of CRRT initiation, and the detailed information of CRRT prescription, were not considered in the analyses. These unidentified factors may have interacted with the preset relationships. The study results were not validated in an independent cohort. The cause of death could not be obtained in the present dataset.

Patients with AKI requiring CRRT are at risk of high mortality, and thus, prediction and alarming of their outcomes throughout monitoring of MAP are critical issues. The present study identified that low MAP at CRRT initiation was associated with high mortality, particularly when it was less than 82.7 mmHg. This target value could serve as a risk classification and a potential therapeutic target in patients with AKI requiring CRRT. In addition, the present results provide the basis of future studies regarding other outcomes such as renal outcome after CRRT.

## Supplementary Information

**Additional file 1: Table S1.** Baseline characteristics in the high (≥ 82.7 mmHg) and low (< 82.7 mmHg) mean arterial pressure groups. **Figure S1.** Flow diagram of the study populations.

## Data Availability

The datasets used and/or analyzed during the current study available from the corresponding author on reasonable request.
