# Supplementary material for: Target value of mean arterial pressure in patients undergoing continuous renal replacement therapy due to acute kidney injury
Source: BMC Nephrol. 2021 Jan 9;22:20. doi: 10.1186/s12882-020-02227-4 (PMC7796677; doi:10.1186/s12882-020-02227-4)
Supplement: Supplementary file 1 — Additional file 1: Table S1. Baseline characteristics in the high (≥ 82.7 mmHg) and low (< 82.7 mmHg) mean arterial pressure groups. Figure S1. Flow diagram of the study populations. [file 12882_2020_2227_MOESM1_ESM.docx]

Table S1. Baseline characteristics in the high (≥ 82.7 mmHg) and low (< 82.7 mmHg) mean arterial pressure groups

| Variables | High MAP  (n = 925) | Low MAP  (n = 1,286) | *P*-value |
| --- | --- | --- | --- |
| Age (year) | 64.0 ± 15.3 | 65.7 ± 15.0 | 0.011 |
| Male (%) | 62.8 | 59.4 | 0.106 |
| Body weight (kg) | 61.7 ± 12.7 | 60.4 ± 12.7 | 0.022 |
| Diabetes mellitus (%) | 25.5 | 28.3 | 0.145 |
| Hypertension (%) | 26.6 | 26.4 | 0.935 |
| Ischemic heart disease (%) | 10.5 | 11.4 | 0.520 |
| Chronic obstructive pulmonary disease (%) | 3.9 | 3.5 | 0.628 |
| Cancer (%) | 33.1 | 35.1 | 0.313 |
| Mechanical ventilator (%) | 78.4 | 79.6 | 0.476 |
| Vasopressors (%) | 67.7 | 70.0 | 0.247 |
| Mean arterial pressure (mmHg) | 96.7 ± 12.3 | 69.3 ± 9.6 | < 0.001 |
| Heart rate (/min) | 105.9 ± 25.8 | 103.7 ± 25.9 | 0.049 |
| Respiratory rate (/min) | 23.7 ± 8.0 | 23.3 ± 7.7 | 0.200 |
| Body temperature (°C) | 36.5 ± 1.2 | 36.4 ± 1.7 | 0.304 |
| Laboratory findings |  |  |  |
| pH | 7.3 ± 0.1 | 7.3 ± 0.1 | 0.102 |
| AaDO_2_ | 282.7 ± 196.0 | 297.5 ± 199.1 | 0.162 |
| White blood cells (×10^3^/uL) | 16.7 ± 23.6 | 17.9 ± 30.8 | 0.315 |
| Hemoglobin (g/dL) | 9.9 ± 2.2 | 9.7 ± 2.1 | 0.002 |
| Platelet (×10^3^/uL) | 114.1 ± 83.8 | 117.3 ± 93.2 | 0.501 |
| Sodium (mmol/L) | 138.3 ± 7.1 | 138.5 ± 7.8 | 0.514 |
| Potassium (mmol/L) | 4.4 ± 1.0 | 4.4 ± 1.0 | 0.985 |
| BUN (mg/dL) | 50.7 ± 29.3 | 50.3 ± 30.5 | 0.729 |
| Creatinine (mg/dL) | 3.0 ± 2.3 | 2.8 ± 2.0 | 0.025 |
| Albumin (g/dL) | 2.9 ± 0.6 | 2.7 ± 0.6 | < 0.001 |
| Bilirubin (mg/dL) | 4.4 ± 7.3 | 4.4 ± 7.3 | 0.953 |
| CRRT dose (ml/kg/hr) | 41.0 ± 12.8 | 41.9 ± 13.6 | 0.148 |
| APACHE II | 21.3 ± 9.5 | 23.2 ± 9.8 | < 0.001 |

AaDO_2_, alveolar-arterial oxygen difference; APACHE, Acute Physiology and Chronic Health Evaluation; CRRT, continuous renal replacement therapy; MAP, mean arterial pressure.

Figure S1. Flow diagram of the study populations


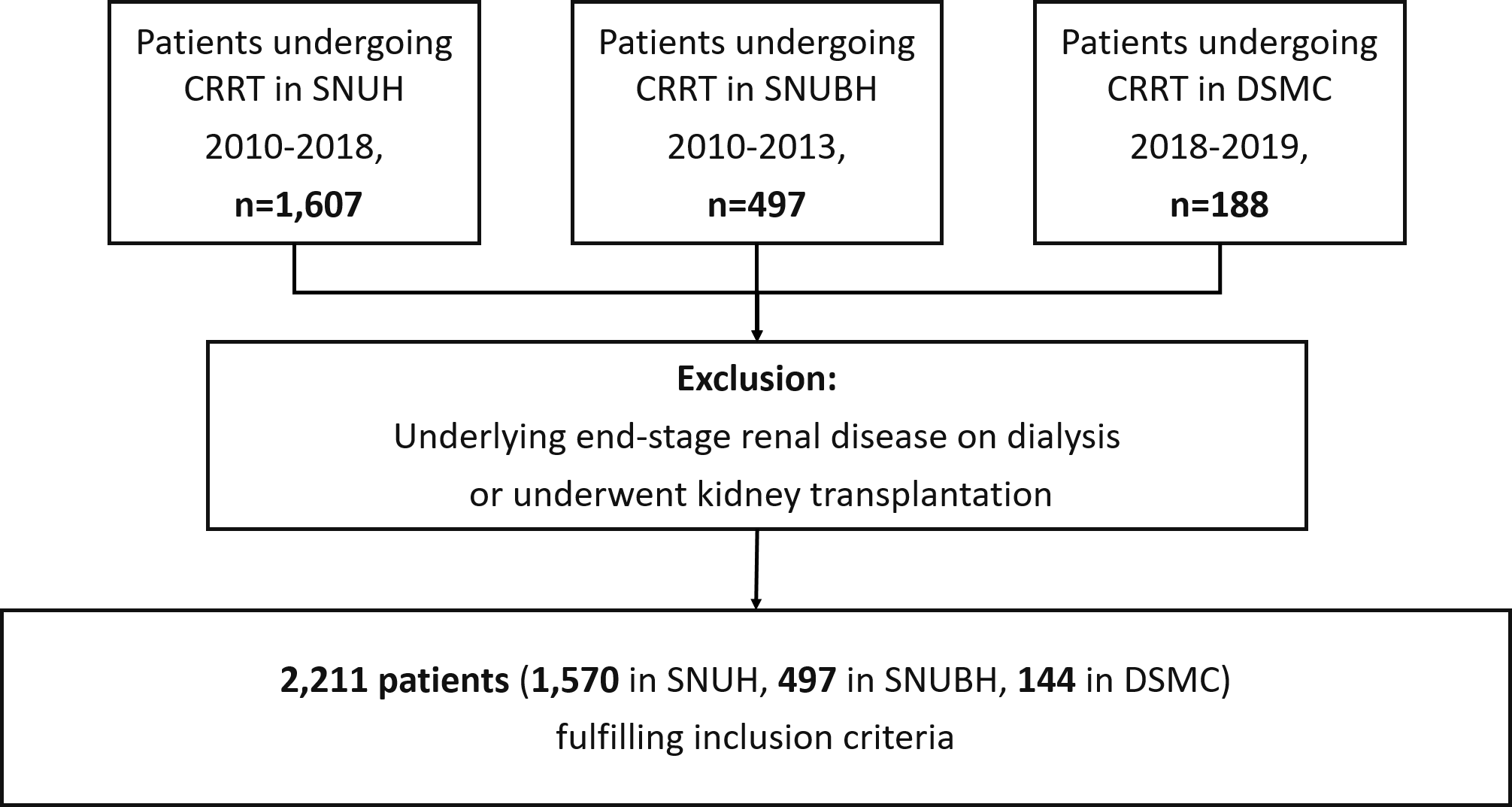


CRRT, continuous renal replacement therapy; SNUH, Seoul National University Hospital; SNUBH, Seoul National University Bundang Hospital; DSMC, Dongsan Medical Center
